# Potential Role of Phytochemical Extract from Saffron in Development of Functional Foods and Protection of Brain-Related Disorders

**DOI:** 10.1155/2022/6480590

**Published:** 2022-09-22

**Authors:** Zahra Maqbool, Muhammad Sajid Arshad, Anwar Ali, Afifa Aziz, Waseem Khalid, Muhammad Faizan Afzal, Sneh Punia Bangar, Mohamed Addi, Christophe Hano, Jose Manuel Lorenzo

**Affiliations:** ^1^Department of Food Science, Government College University, Faisalabad, Pakistan; ^2^Department of Epidemiology and Health Statistics, Xiangya School of Public Health, Central South University, China; ^3^Department of Food, Nutrition and Packaging Sciences, Clemson University, Clemson 29634, USA; ^4^Laboratory of Improvement of Agricultural Production, Biotechnology and Environment, Department of Biology, Faculty of Sciences, Université Mohamed Premier, Oujda 60000, Morocco; ^5^Department of Biochemistry, Plant Natural Products Lab, University of Orleans, Chartres, France; ^6^Centro Tecnológico de la Carne de Galicia, Adva. Galicia N° 4, Parque Tecnológico de Galicia, San Cibrao das Viñas, 32900 Ourense, Spain; ^7^Universidade de Vigo, Área de Tecnología de los Alimentos, Facultad de Ciencias de Ourense, Universidad de Vigo, 32004 Ourense, Spain

## Abstract

The present review is designed to measure the effects of saffron extract in functional foods and its pharmacological properties against various disorders. Saffron is a traditional medicinal plant used as a food additive. The stigma of saffron has bioactive compounds such as safranal, crocin, crocetin, picrocrocin, kaempferol, and flavonoid. These bioactive compounds can be extracted using conventional (maceration, solvent extraction, soxhlet extraction, and vapor or hydrodistillation) and novel techniques (emulsion liquid membrane extraction, ultrasound-assisted extraction, enzyme-associated extraction, pulsed electric field extraction, microwave-assisted extraction, and supercritical fluid extraction). Saffron is used as a functional ingredient, natural colorant, shelf-life enhancer, and fortifying agent in developing different food products. The demand for saffron has been increasing in the pharma industry due to its protection against cardiovascular and Alzheimer disease and its antioxidant, anti-inflammatory, antitumor, and antidepressant properties. Conclusively, the phytochemical compounds of saffron improve the nutrition value of products and protect humans against various disorders.

## 1. Introduction

The blooming perennial plant *Crocus sativus* L. (family Iridaceae) has been farmed in southern Europe, India, and Iran for millennia under certain climatic circumstances [1]. Saffron is one of the costliest culinary plants, used as a preservative, food component, coloring agent, and traditional medicine. It is prepared from the dried stigmas of the flower. Stigma collecting begins with picking flowers a few days after they open in the autumn. The stigmas of saffron are then manually plucked from flowers, which impacts the quality and price of saffron [2]. Saffron is a rich source of different bioactive chemicals with noticeable antioxidant effects due to the chemical composition of stigmas, which are related to primary and secondary metabolites. The most prominent ones contributing to saffron sensory qualities are crocin, crocetin, picrocrocin, and safranal. Saffron spice stigmas have a high antioxidant capacity [3]. Saffron has been prized in various cuisines for its unusual color, great flavor, and distinct scent. The sugar esters of crocetin (apocarotenoids) are extremely soluble in water, specifically designated as crocin, causing yellow color. Carotenoids, carotenes, zeaxanthin, and lycopene are among the nonvolatile components [4]. The extraction of bioactive components from saffron necessitates a continuous search for economically and environmentally viable extraction strategies. Traditional extraction procedures take a long time and require a large amount of solvent. Novel extraction strategies for extracting bioactive components from saffron have been developed, reducing extraction time and solvent usage while improving extract quality and yield [5]. There are wide and traditional medicinal uses of saffron in Tibetan medicine, Persian medicine, Ayurvedic medicine, and traditional Chinese medicine; and also, been used as an antibacterial, antidepressant, digestive, anticancer, and anticonvulsant [6]. Mental illnesses and neurological and neurodegenerative disorders are a major burden because of their danger of impairment, rising frequency, and lack of effective causal/disease-modifying treatments [7]. Due to their high incidence, which continues to climb in tandem with increasing life expectancy and their accompanying disabilities, lack of effective and tolerable therapies, and enormous economic burden, brain illnesses pose a serious threat to human health. Cardiovascular diseases, mental health issues, and neurological disorders have become major contributors to the worldwide burden of noncommunicable diseases as the population ages (NCDs) [8]. Saffron extract and its active components have been the subject of several pharmacological investigations for possible therapeutic implications in cancer, depression, and memory loss.

Saffron is now mostly utilized for its antioxidant effects and is present in various dietary supplements. Saffron has a bitter flavor and gives meals a bright yellow-orange color. Saffron is commonly utilized in Persian, Arab, Central Asian, European, Indian, Moroccan, and Cornish cuisines because of its unique flavor and color. Saffron is often used in confectioneries and liquors [9]. Several reviews and mini-review studies on the biological activity of saffron and its compounds have been published regarding its pharmacological qualities and potential therapeutic applications. Parallel to this, the potential uses of saffron and its active constituents have prompted an increase in research studies, some of which have yielded some very useful and intriguing results of active saffron ingredients, particularly in the fight against cancer, cardiovascular disease, and Alzheimer disease [9]. In this review, we want to describe and critically evaluate prior and current findings on the biological/pharmacological actions of saffron and its active components, as well as their potential therapeutic applications. Following that, a thorough and critical evaluation of existing preclinical and clinical research and review articles on the pharmacology and possible therapeutic uses of saffron and its key active components will be presented.

## 2. Overview of the Phytochemicals from Saffron

Phytochemicals are physiologically active, naturally occurring chemical compounds found in plants that have health benefits for humans beyond macronutrients and micronutrients [10]. Phytochemicals can be found in various plant parts, including the roots, stems, leaves, flowers, fruits, and seeds.

Saffron has been grown for flower and metabolites from the dried stigma. Picrocrocin, kaempferol, safranal, phenol, delphinidin, flavonoid, and crocetin are the primary phytochemicals in saffron; and they have good quality bioactivity and antioxidant potential [5]. After chemical analysis, fat, carbohydrates, minerals, vitamins, and other secondary metabolites such as anthocyanins, carotenoids, flavonoids, and terpenes have been discovered in saffron stigmas [11]. Stigma contains bioactive elements such as anthocyanins, pigments, flavonoids, volatile fragrant essences, and vitamins [12]. According to a chemical study, over 150 volatile and nonvolatile chemicals have been discovered in saffron stigmas [13]. Safranal, crocetin, and picrocrocin esters are three main chemicals found in saffron. Carbohydrates, raw fiber, proteins, lipids, anthocyanins, minerals, carotenoids, flavonoids, and a variety of other nutrients are also present. These bioactive compounds are helpful in human health [12].

Saffron is utilized in traditional recipes for its flavor, color, and aroma, despite the biological effects and chemical qualities of significant bioactive chemicals present in saffron. It is not only used as a spice, but it has long been renowned as a medicinal plant due to its therapeutic potential. The emergence of synthetic chemistry-based medications impacted saffron medical and pharmacological applications [14]. All bioactive chemicals in saffron have anticancer, antioxidant, antidepression, and antitumor properties and reduce insomnia and anxiety [15, 16]. The phytochemical composition of saffron is listed in Table 1.

### 2.1. Carotenoids

These are the natural pigments present in pigments of fungus, algae, plants, and animals in the form of yellow hues, orange-red, and red. Carotenoids are poly isoprenoid substances that fall into oxygenated hydrocarbon derivatives (xanthophylls) and hydrocarbon carotenoids (carotenes). A conjugated double bond system is a structural feature that determines its chemical, biological, and physical characteristics [26]. Saffron is a famous culinary coloring agent due to its high carotene concentration. Carotenoids of saffron have a variety of bioactivities, including antioxidant, anti-inflammatory, and immunomodulatory properties [27]. Dietary carotenoids must be free from the food matrix and integrated into mixed micelles mixtures of bile salts and many lipids to be absorbed in the intestine. As a result, the fat in a meal is required for carotenoid absorption. Carotenoid supplements in oil liberated from the matrix are easier to absorb than carotenoids in meals [28]. Carotenoids are integrated into chylomicrons, triglyceride-rich lipoproteins, and released into the blood by intestinal cells (enterocytes). The action of an enzyme called lipoprotein lipase depletes triglycerides from circulating chylomicrons, resulting in the production of chylomicron remnants. Provitamin A (carotenoids) can be cleaved in the gut and liver to create retinal pigment [28]. Carotenoids have been demonstrated to offer two health benefits: improved immunological response and a lower chance of degenerative illnesses such as cancer, cardiovascular disease, cataracts, and muscle degeneration [15].

### 2.2. Safranal, Crocins, and Picrocrocin

Crocins are used in the food industry to provide a bright red color that is stable and water-soluble. Their antioxidant characteristics make them effective memory boosters, antischizophrenia, and anti-Alzheimer disease. Crocetin and its derivatives are also utilized as colorants in the pharmaceutical and food industries and have a variety of health-promoting medicinal benefits [12, 29]. Picrocrocin is the colorless part of the stigma that gives it its bitter taste and aroma. The volatile chemicals including isophorone and safranal to contribute the saffron aroma. Anticonvulsant and antidepressant properties of isophorone and safranal are well-known [13].

Crocetin, crocin, and picrocrocin have been shown the cancer-preventive, memory-enhancing, and heart-protective characteristics. The major sources of saffron color are crocin and crocetin, whereas the bitter flavor is due to picrocrocin. Saffron is used as a medication or in health goods. The crocin content of saffron is an important quality measure [30]. Picrocrocin and safranal are two more physiologically active phytoconstituents found in saffron derived from zeaxanthin degradation. Picrocrocin is responsible for flavor and bitterness, whereas safranal gives saffron its distinct scent. Saffron essential oil contains picrocrocin, which is a monoterpene glycoside. The overall output of saffron essential oil might range between 0.4 and 1.3%. It is the second most prevalent phytochemical in saffron essential oil with its bitter taste and flavor. It accounts for about 1 to 13% of the dry weight of saffron [31]. Safranal is a monoterpene aldehyde found in saffron essential oil. Safranal is formed by the action of *β*-glucosidase on its precursor, picrocrocin, during postharvest dehydration, and storage conditions on fresh stigmas of *C. sativus* [32].

### 2.3. Flavonoids

Flavonoids are secondary polyphenol metabolites found in a wide range of plants and diets [33]. Secondary metabolites of plants have antiproliferative activities, antitumor, proapoptotic, antioxidant, cardioprotective, and anti-inflammatory [34]. Corresponding glycosides and flavanols that can be used as food additives are present in flavonoids. They also show cardiac protective effects. Three primary kaempferol glucosides have been found in *C. sativus* stigmas.

All the activities of flavonoids in plants include UV protection, flower coloration to attract pollinators, allelopathy, defense, control of reactive oxygen species, plant-microbe communication, and auxin transport inhibition. They are also necessary for pollen viability in many species [35]. Flavonoids impact various biological processes, including cell-to-cell communication, transcriptional control, and signal transduction. Furthermore, flavonoids play an important role in human nutrition, and various therapeutic effects of flavonoids have been discovered in animal systems [36].

### 2.4. Monoterpenes, Monoterpenoid Derivatives, Amino Acids, and Alkaloids

Isoprene and its oxygenated and saturated derivatives are polymerized from two molecules of isoprene that result in the formation of terpenoids that are usually referred to as monoterpenes. Monoterpene glycosides and monoterpenoids that have been identified and isolated from saffron are 38 in number. *C. sativus* stigmas also contain nitrogen substances, such as alkaloids and amino acids. Amino acids contribute to the flavor of foods and are essential components of food ingredients [37]. Alkaloids are antibacterial, anti-inflammatory, and antiviral, exhibiting pharmacological activity [38].

### 2.5. Phenolic Acids

The most common group of bioactive chemicals found in various plant sources is phenolic acids [39]. The primary phenolic acids include hydroxycinnamic acids and hydroxybenzoic acids, which are secondary aromatic metabolites that add typical organoleptic features to food and are associated with various health advantages [40]. Chlorogenic acid, caffeic acid, methylparaben, gallic acid, and pyrogallol are phenolic acids that have been isolated and discovered in saffron [41]. Hydroxybenzoic acids (flavonoid biosynthesis precursors) have been found in several sections of *C. sativus*. p-hydroxybenzoic acid, h-coumaric acid, sinapic acid, and vanillic acid are among the hydroxycinnamic acids found in saffron petals [42], whereas *p*-hydroxybenzoic acid and benzoic acid were also identified in *C. sativus* pollen [43].

### 2.6. Phytosterols

Phytosterols are naturally occurring steroid alcohols that have good nutritional and wellness effects by decreasing blood cholesterol, and the ratio of low-density lipoprotein (LDL) bound cholesterol in serum [44]. In *C. sativus*, phytosterols such as stigmasterol and *β*-sitosterol were found, while fecosterol, stigmasterol, and *β*-sitosterol were found in the petals [45].

## 3. Conventional and Novel Extraction Techniques of Saffron Bioactive Compounds

Bioactive components can be extracted from plant materials by considering the relevant tissues and plant varieties and using the right extraction process [46]. Traditional extraction processes (maceration, solvent extraction, soxhlet extraction, and hydrodistillation) are often nonselective, need longer extraction times, employ a high volume of organic solvents, and in some cases, damage heat-sensitive bioactive chemicals [47]. Novel extraction techniques are recommended as alternatives to traditional methods to address these issues. These “green” extraction processes are environmentally benign, faster, safer, more efficient, and precise.

**Figure 1:**
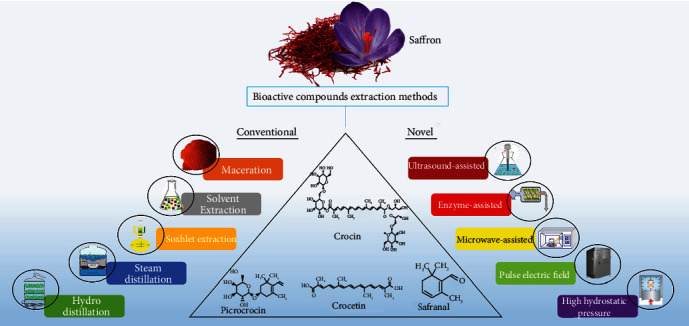
Different conventional and novel extraction methods used to extract the bioactive compounds.

Green approaches include emulsion liquid membrane extraction, ultrasound-assisted extraction, enzyme-associated extraction, pulsed electric field extraction, microwave-assisted extraction, and supercritical fluid extraction [46, 48]. These methods can efficiently extract saffron bioactive components. In general, the effectiveness of extraction procedures is largely determined by the use of coextraction techniques, choice of appropriate solvents, and consideration of solvent-solute affinity [49]. Extracting, isolating, and characterizing these bioactive elements are difficult because bioactive chemicals are temperature, light, and humidity sensitive [50].  Figure 1 shows an overview of the conventional and novel methods of extracting the bioactive compounds from saffron.

## 4. Saffron Fortification in Food Products

Saffron is an important spice and one of the most researched functional foods, made from the dried stigmas of *C. sativus*. It is a major source of crocins (carotenoid derivatives), safranal, and picrocrocin. However, these compounds have vital nutraceutical and medicinal effects. The color, bitter flavor, and aroma imparted to meals are primarily utilized in cooking [51].

The antioxidant, microbiological, physicochemical, and sensory aspects of yogurt fortified with saffron were investigated by Raimondo et al. [52]. During storage, the impact of saffron on the properties of yogurt was studied. According to sensory analysis data, color, odor, and texture, all had a consistent impact on the acceptability of the saffron enriched yogurt. According to sensory analysis data, color, odor, and texture, all had a consistent impact on the acceptability of the saffron enriched yogurt. The inclusion of saffron dramatically boosted antioxidant activity, indicating that saffron fortification of yogurt results in a novel fermented product that can be used to complement antioxidant intake.

Ahmad et al. [53] improved the nutraceutical potential by fortifying the Himalayan cheese (grade) with saffron. The goal of the study was to make saffron-enhanced Kradi cheese. The physicochemical, antioxidant, and therapeutic qualities of the product were investigated. The enriched Kradi had much higher “a” and “b” values than the control. The total phenolic content and antioxidant activity of enriched Kradi were increased significantly due to secondary saffron metabolites. When compared to the control, the enhanced Kradi had better organoleptic qualities.

Ahmad et al. [54] demonstrated the development of functional biscuits. As a natural antioxidant source, saffron extracts in two strengths were synthesized and employed in whole wheat flour cookies. Over a 9-month storage period, the influence on the product color, texture, and sensory qualities was also investigated. When saffron extracts were added to cookies, the quality of the cookies greatly improved. After saffron extracts were added to cookies, they demonstrated outstanding antioxidant qualities. Saffron powerful antioxidant action and the stability of its extracts while baking were credited with this. Compared to control cookies, cookies with saffron extract were ranked top for all sensory attributes except texture. It can be inferred that adding saffron extract is a natural antioxidant to cookies and can make them healthier. Furthermore, natural antioxidants are harmless and can be employed to extend the stability of bakery items with high fat/oil.

The dry stigma of saffron accumulated both volatile and nonvolatile chemicals that assist improve food aroma and quality. The dry stigmas of the flowers contain active chemicals such as picrocrocin, crocin, and safranal. Saffron is used to treat various illnesses and is also prized for its color, flavor, and aroma. It is used as a colorant for a variety of things. Saffron has been associated with increasing immunological response. Crocins have been shown to have antiapoptotic, anti-inflammatory, and antioxidant properties. Ali [55] proved that saffron is a natural organic colorant that is quite durable. It is primarily employed for its great coloring strength. To meet the potential needs of consumers, applied research methodologies are important for producing healthy saffron products. As a result, it has a wide range of uses in aquatic conditions, and its use in the food business is relatively inexpensive.

**Figure 2:**
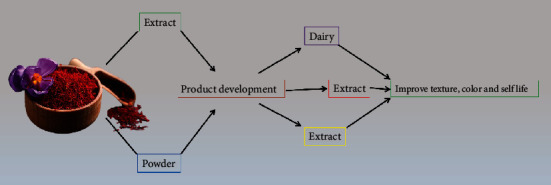
Functional behavior of saffron in the development of food products.

The physicochemical, rheological, antioxidant, sensory, and survival aspects of a probiotic saffron-based beverage were examined during the fermentation process by Arasb et al. [56]. During the fermenting process, the antioxidant capacity of samples increased significantly, whereas total anthocyanin concentration declined significantly. In comparison to other strains, *Lactobacillus casei* lived much longer in the fermented extract. According to the findings, saffron-based beverage provides an appropriate medium for lactic acid bacteria development to manufacture useful beverages. The functional behavior of saffron in developing food products is listed in Figure 2.

## 5. Therapeutic Properties of Saffron to Protect Different Disorders

*C. sativus* is widely utilized in tropical and subtropical areas for domestic and medical purposes. The plant stigmas are employed because they contain several chemical ingredients such as crocetin, crocin, and other flavonoids that provide a wide range of therapeutic benefits for treating various diseases. From prehistoric times, saffron has been used in traditional medicine [57] (Table 2).

### 5.1. Improve Cardiovascular Performance

The leading cause of death worldwide is cardiovascular disease (CVD). CVD refers to various conditions affecting the heart muscle and the circulatory system that supplies the heart, brain, and other essential organs. The epidemiological shift has elevated cardiovascular disease to the world leading cause of death [65].

Saffron has been proven in several studies to control cardiovascular function, maintain fundamental vascular tension, and play a significant role in maintaining the circulatory system steady state. By reducing oxidative stress and dyslipidemia, crocin in saffron may help to avoid diabetes-related cardiovascular problems [66]. The previous study found that by extending the effective refractory time of cardiac cells, saffron can minimize the occurrence of catastrophic ventricular arrhythmias after reperfusion in rats [67]. Damage in rabbit hearts and doxorubicin-induced cardiac dysfunction can be improved by scavenging free radicals, maintaining antioxidant enzymes, and reducing lipid peroxidation [68]. Both crocetin and crocin are in saffron and have endothelium-dependent prorelaxing and procontractile properties that work through smooth muscle cell pathways. Saffron has some antivasoconstriction properties in hypertension [69], thus possibly reducing blood pressure. By aortic remolding and limiting blood pressure rise, saffron was shown to the medium thickness of the elastic lamellae and to lower the cross-sectional area of the aorta [70]. Another study found a considerable decrease in total cholesterol and plasma levels of triacylglycerol due to crocin extract from saffron [71].

### 5.2. Antioxidant Activity

Antioxidants can help prevent cancer, aging, and other disorders by inhibiting free radical oxidation. Many researchers have discovered the antioxidant effects caused by saffron extracts and secondary metabolites [72]. Crocin, picrocrocin, and safranal in saffron have significant activity against oxidation, while radical scavenging action has been observed in safranal, picrocrocin, and crocin [73]. Greater quantities of antioxidant activity and total polar phenols than other spices are exhibited by saffron flower by-products [74]. Free radicals can be captured by safranal and crocin, but crocetin can efficiently reduce lipid peroxidation and eliminate free radicals. They have the potential to be employed to prevent cancer and treat cardiovascular and psychological diseases [27]. The ethanolic and aqueous extracts of saffron have antioxidant effects. Saffron ethanolic extracts scavenge hydroxyl radicals and accelerate deoxyribose breakdown, whereas aqueous and ethanolic extracts inhibit malondialdehyde formation and lipid peroxidation in red blood cells [75].

Furthermore, the antioxidant action of saffron has been demonstrated in vivo investigations in asthmatic mice bronchial epithelial cells [76]. The main component of saffron is crocin that has been shown to protect brains, livers, and kidneys from oxidative damage caused by continuous restraint stress [77]. An aqueous saffron extract possesses the capacity to prevent the activation of reactive oxygen species and intracellular signals by boosting cell viability and inhibiting the apoptotic pathway and antioxidant activity [78]. Excess-free radical production has been linked to cancer, cardiovascular disease, aging, diabetes, and other illnesses [79].

### 5.3. Anti-Inflammatory Properties

Acute inflammation is the immune system defensive reaction to invading pathogens or tissue damage. If not treated, acute inflammation can lead to organ diseases and chronic inflammatory phenotypes [80].

Saffron extracts include a variety of antioxidant chemicals (such as crocins, crocetin, quercetin, and kaempferol) that can prevent the generation of proinflammatory cytokines in various animal models and thus has anti-inflammatory qualities [81]. Similarly, Somayyeh et al. [82] found that white blood cell count, neutrophil count, eosinophil count, and platelet number in the blood of ovalbumin-sensitized rats can enhance by hydroalcoholic saffron extracts. In rats, hydroethanolic extracts of saffron have been demonstrated to protect against ischemia and acute kidney disorder [83]. Saffron has anti-inflammatory properties because it contains alkaloids, anthocyanins, saponins, tannins, and flavonoids [27].

### 5.4. Antitumor Properties

Cancer is a group of disorders characterized by uncontrollable cell division [84]. Numerous studies have demonstrated saffron extract suppresses cancer cell growth and tumor formation. Saffron extracts can stop tumor cells from growing [85]. In human cancer cell lines in vitro, saffron carotenoids have inhibited cell proliferation [86]. Crocetin and crocin can inhibit colorectal cancer cell proliferation and invasion [87] and reduce the size of tumors [88]. Encapsulation of crocin in saffron can protect against colon cancer [89]. The downregulation of metalloproteinases and urokinase by saffron extracts crocetin and crocin inhibit invasion and migration of prostate cancer cells. Crocetin has a higher anticancer impact, restoring epithelial-mesenchymal transition by decreasing N-cadherin and *β*-catenin expression while raising E-cadherin expression [90]. Due to carotenoids interactions with topoisomerase II, its effects on cellular RNA or DNA production to inhibit free radical chain reactions saffron and its constituents may have anticancer properties [91]. According to research, various diseases including hepatic cancer, colorectal cancer, gastric cancer, pancreatic cancer, ovarian cancer, cervical cancer, prostate cancer, lungs cancer, skin cancer, breast cancer, and leukemia can be treated by saffron [92].

## 6. Therapeutic Benefits of Saffron in Brain Diseases

**Figure 3:**
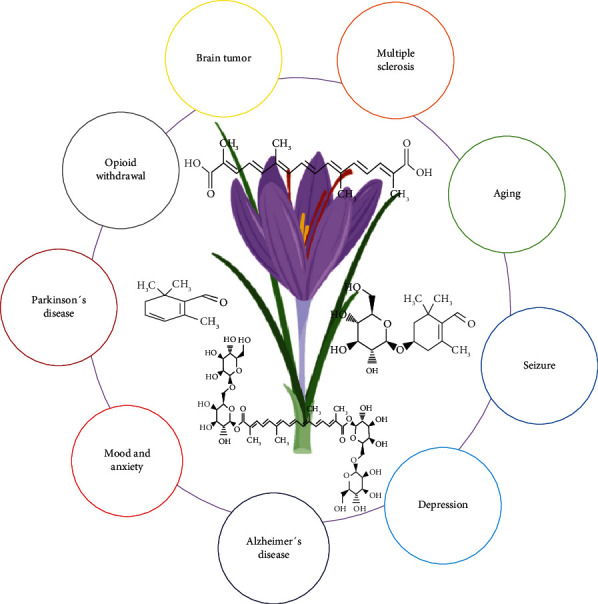
Schematic overview of the main therapeutical benefits of saffron associated with brain diseases.

Saffron has been used to treat different brain-related disorders. In human clinical trials as well as in animal studies, it has been proved that saffron has reduced the level of different brain diseases (Figure 3).

### 6.1. Antidepressant Effects

Depression is a prevalent condition that significantly impacts psychosocial functioning and quality of life [93]. Saffron has turned to traditional Chinese herbal medicine to avoid some side effects in patients suffering from depression due to chemotherapy treatment. In several recent investigations, saffron extracts have been shown to have antidepressant properties [16, 94]. According to a study, immediate treatment of rats with crocin and crocetin from saffron exhibited antidepressant effects in a forced swimming test. Oral administration of large dosages of crocin and crocetin in the forced swimming and tail suspension tests has reduced immobility time while not affecting the mice movement and coordination [95]. From saffron stigmas, safranal and crocin may play a role in the antidepressant effect. Crocin can block dopamine and norepinephrine uptake, while safranal may block serotonin uptake [96]. In treating mild to moderate depression in both in vivo and in vitro trials, saffron has proven effective. Compared to antidepressants, saffron had a better therapeutic impact in placebo-controlled trials. Saffron antidepressant properties are thought to stem from its antioxidant, serotonergic, neuro-endocrine, neuroprotective, and anti-inflammatory properties [97].

According to several recent clinical investigations, Saffron appears to have an antidepressant effect. A six-week saffron capsule was given to patients suffering from moderate depression in a parallel-controlled, double-blind, randomized research. Saffron efficiently treated mild to moderate depression [98]. To diminish the symptoms of depression with time, saffron capsules were shown in a clinical investigation with elders. For seniors who are hesitant to utilize synthetic medications, saffron could be a strong antidepressant [99]. During clinical investigations, it was proved that saffron can be proved helpful for treating mild to moderate depression in adults and older people. Different clinical findings suggested that to treat serious depression, saffron might be used safely [100]. Aside from the antidepressant impact, in type 2 diabetic patients, sleep disturbance and anxiety were also considerably reduced due to saffron [101]. In general, saffron has a good impact on the treatment of depression [102].

### 6.2. Alzheimer Disease

Alzheimer disease is a common type of senile dementia and a central nervous system degenerative illness. The most common symptoms are cognitive dysfunction, personality changes, progressive memory impairments, language obstacles, and neuropsychiatric. Worldwide, it is the frequent cause of dementia, and its incidence is on the rise in the world aging population. The pathologies of amyloid plaque formation and hyperphosphorylated neurofibrillary tangles are two prominent pathologies of this neurodegenerative disease process. The clinical presentation that meets numerous criteria and fluid and imaging indicators is used to make a diagnosis [103]. Saffron extracts, particularly trans-crocetin, lower A42 in monocytes in Alzheimer patients [104]. Amyloid aggregation and deposition in the human brain can be prevented by saffron. In double-blind placebo-controlled research, few patients with Alzheimer disease were randomized to receive saffron capsules. Psychometric measures revealed that patients treated with saffron improved cognitive function after 16 weeks. In adverse event reports between the two groups, there was no significant difference proving that saffron is safe and effective in treating mild to moderate Alzheimer disease [105].

### 6.3. Anxiety Disorders

Anxiety is the most common mental disorder associated with a high burden of illness that is generalized by anxiety, panic disorders, and others [106]. The most prevalent type of neurosis is anxiety. Clinical research has proven the usefulness of saffron in lowering anxiety [107]. Few diabetic patients were given either 300 milligrams of saffron per week or a placebo. Using the Spielberger State-Trait Anxiety Inventory scale, participants' anxiety levels were assessed. The findings revealed that saffron could successfully lower diabetes patients' anxiety [108]. In another study, 60 anxiety sufferers were randomly assigned to receive a placebo pill or 50 mg of saffron twice a day for twelve weeks. In the final week, saffron proved a substantial effect for anxiety treatment compared to placebo [109].

### 6.4. Anti-Parkinson Effects

In animal models of neurodegenerative disorders, saffron and its constituents (primarily crocin, crocetin, and safranal) have been employed [22]. Crocin and safranal both suppress *apo*-*α*-lactalbumin (*a*-*α*-LA) fibrillation under amyloidogenic circumstances, with crocin being more effective than safranal [110]. The formation of harmful amyloid structures has been linked to several neurodegenerative illnesses, including Alzheimer and Parkinson diseases [111].

Crocetin (25, 50, and 75 g/kg body weight) has been shown to protect rats against 6-hydroxydopamine- (6-OHDA-) induced Parkinson disease after a seven-day treatment. A plausible explanation was postulated as a tissue decrease in dopamine consumption [112]. A mouse model of acute MPTP- (1-methyl-4-phenyl-1,2,3,6-tetrahydropyridine-) induced Parkinson disease was used to investigate the protective impact of saffron pretreatment on dopaminergic cells in the substantia nigra pars compacta (SNc) and retina. Over 30 hours, BALB/c mice were given MPTP or saline. Saffron (0.01 percent *w*/*v*) dissolved in drinking water was given to animals in the saffron-treated group for five days, whereas control groups got standard tap water. After six days, the brains were processed for tyrosine hydroxylase (TH) immunochemistry, and the number of TH^+^ cells was counted using the optical fractionator technique. MPTP-injected animals had fewer TH^+^ cells (30-35%) in both the SNc and retina than saline-injected controls. Saffron pretreatment of MPTP-injected mice boosted SNc and retinal TH^+^ cell counts by 25-35%, bringing them back to normal levels. According to the findings, saffron pretreatment rescued many dopaminergic cells in the SNc and retina against Parkinsonian (MPTP) damage in mice [113].

### 6.5. Effects on Neuroinflammation

Crocin decreased neuropathology and prevented syncytin-1 and nitric oxide- (NO-) induced astrocyte and oligodendrocyte cytotoxicity in experimental autoimmune encephalomyelitis (EAE) with much fewer neurological deficits [114]. Syncytin-1 has been linked to neuroinflammation and oligodendrocyte death. In multiple sclerosis lesions, syncytin-1 is strongly expressed in astrocytes, microglia, and glial cells. Stress in the endoplasmic reticulum (ER) has been linked to inflammatory pathways. EAE has been demonstrated to enhance the expression of the ER stress genes XBP-1/s [115]. Crocin administration on day 7 after EAE induction decreased the expression of ER stress genes XBP-1/s and repressed ER stress and inflammatory gene expression in the spinal cord [116].

### 6.6. Effects on Brain Neurotransmitters

According to studies, saffron aqueous extract (50, 100, 150, and 250 mg/kg) enhanced brain dopamine levels in a dose-dependent way. Furthermore, the extract showed no influence on serotonin or norepinephrine levels in the brain [115, 117]. Furthermore, the findings revealed that the aqueous extract of saffron, at a concentration of 250 mg/kg, activated and boosted the synthesis of essential neurotransmitters such as dopamine and glutamate in the rat brain [117].

### 6.7. Effects on Oxidative Damages and Neurotoxicity

Crocin 10 M was shown to limit the generation of peroxidized lipids in cultured PC12 cells, moderately restore superoxide dismutase (SOD) activity, and preserve the shape of neurons. Crocin antioxidant impact was equivalent to tocopherol, but it was considerably stronger at certain doses.

In rats, administration of *C. sativus* stigma extract (100 mg/kg) for 7 days before induction of cerebral ischemia by middle cerebral artery occlusion (MCAO) significantly lowered SOD, catalase, and Na/K-ATPase activities, as well as glutamate and aspartate concentrations [116]. In PC12 cells, treatment with saffron extract (5 and 25 mg/mL) and crocin (10 and 50 M) reduced the neurotoxic impact of glucose [117]. The findings revealed that glucose (13.5 and 27 mg/mL) decreased PC12 cell viability, whereas saffron and crocin pretreatment reduced cell mortality. Another research found that giving mice saffron extract (200 mg/kg) and honey syrup (500 mg/kg) for 45 days decreased the neurotoxicity caused by aluminum chloride [118]. Other investigations found that safranal protects hippocampus tissue from ischemic rats and hippocampal tissue after quinolinic acid (QA) injection against several indicators of oxidative damage. Following kainic acid injection, safranal lowered extracellular glutamate and aspartate (excitatory amino acids) concentrations in the hippocampus of anesthetized rats [119].

### 6.8. Effects on Neuronal Injury and Apoptosis

Crocin (30, 60, and 120 mg/kg) reduced infarct volume and protect against ischemia/reperfusion damage and cerebral edema in a rat stroke model [120]. Crocin (60 mg/kg) was given one hour before or after the development of ischemia and decreased cerebral edema. Crocetin neuroprotective benefits in animal experiments have been linked to its capacity to block apoptosis early in the injury and enhance angiogenesis later on, as guided by greater levels of vascular endothelial growth factor receptor-2 (VEGFR-2) and serum response factor (SRF) [121]. Crocin (50 mg/kg) reduced RGC apoptosis following retinal ischemia/reperfusion damage through the phosphatidylinositol 3-kinase/AKT (PI3K/AKT) signaling pathway in recent research. Crocin also enhanced the Bcl-2/BAX ratio [122]. Crocin (10 M) suppresses neuronal cell death triggered by both internal and external apoptotic stimuli by suppressing tumor necrosis factor-alpha- (TNF-) driven production of proapoptotic mRNA, which releases cytochrome c from mitochondria. Crocetin may also prevent RGC-5 cell death caused by H_2_O_2_ by inhibiting caspase-3 and caspase-9 activity [123].

## 7. Potential Health Risks of Saffron

Health risk is basically something that enhances the chances of developing a disease. Basically, evolution of farming practices for saffron has been based on an “Organic” system of production. Saffron growers in most parts of world have so far used no agrochemical input for production and most of the inputs used were internal. Agronomic practices including application of organic fertilizers, nonchemical methods for pests and weed control, complete family labor work for production and processing, share-cropping, and sociocultural environment surrounding the whole process of saffron are in compliance with organic farming principles [124]. However, due to this reason, saffron may be free from toxic compounds. In some country, where saffron farming performed using nonorganic source including fertilizers and chemical, it may be containing some toxic compounds that produce serious effect on human health. On the other way, during the handling and processing, it may be produce some toxic compounds due to improper handling and storage. These toxic compounds can react with other compounds and create some serious health problems including oxidative damages, neuronal injury, anxiety, and cancer [125].

## 8. Conclusion and Future Trends

It is concluded that different phytochemicals extracted from saffron have valuable in developing functional food products and have medicinal properties. Saffron is a traditional medicinal plant that contains bioactive compounds. Saffron contained some functional ingredient, natural colorant, shelf-life enhancer, and fortifying agent. These functional ingredients can be extracted using conventional and novel techniques. Both conventional and novel technologies are being used to extract bioactive components from saffron. Various functional foods can be developed using saffron extract as a functional ingredient, natural colorant, shelf-life enhancer, and fortifying agent. These phytochemical compounds extracted from saffron have antioxidant, anti-inflammatory, antitumor, and antidepressant properties that help in protection against various disorders.

Saffron potential involvement in the prevention and treatment of brain related diseases has to be investigated further. The antioxidant profile of saffron, which is high in crocin, may inhibit the oxidation process in different foods. Saffron that contains rich bioactive compounds is likely to provide health benefits. It should also be remembered that functional foods can provide their possibly subtle benefits. To prove that saffron foods have potentially beneficial effects on brain-related diseases or risk factors, rigorous scientific investigation in human studies is needed. Future randomized control trials (RCTs) should seek to compare the effects of a control diet and a saffron intervention diet on biomarkers of brain disease or health outcomes so that evidence of longer-term benefits emerges. The impact of the whole meal, reflecting synergy between components, needs to be assessed because most previous research has focused on extracts and components. Background diet can confound results and make it difficult to attribute effects to the dietary variables of interest; therefore, it is equally important to examine the composition of the background diet. In addition, the impact of saffron-based foods on brain disease must be considered in the context of the overall diet and carefully monitored throughout the study to allow for translation to practice. Unfortunately, studies on saffron are limited in their ability to assign direct antioxidant benefits to saffron because they do not consider metabolic transformations and interactions that affect the bioavailability and biological activity of polyphenols in the body after ingestion. Regardless, studying the effects of diet on cancer is difficult, and clinical trials are problematic for ethical reasons. Nonetheless, information obtained from many types of experimental studies contributes to a more complete understanding of how the saffron nutritional matrix may be beneficial.

## Figures and Tables

**Table 1:** Overview of the main (phyto)chemicals from saffron.

Phytochemical	Active compounds	Authors
Vitamins	A, B1, B2, B6, and C	[17, 18]
Minerals	Calcium, magnesium, iron, phosphorus, and potassium	[19–21]
Carotenoids	*β*-Carotene, *α*-carotene, crocetin, and crocins	[22]
Monoterpene	Safranal and picrocrocin	[23, 24]
Isophorones	Isophorone	[25]

**Table 2:** Pharmaceutical properties of saffron.

Compounds	Bioactive constituent	*In vivo/in vitro*	Pharmacological functions	References
Carotenoid	Crocin	Mice	Neuroprotective	[22]
	Crocin and crocetin	Rat	Neuroprotective	[58]
	Crocetin	Human	Antifatigue	[59]
	Crocin	—	Anti-Alzheimer	[60]
	Crocin	Human	Antischizophrenia	[61]
Monoterpene aldehydes	—	Human	Antidepressant	[23]
	Safranal	Mice	Anticonvulsant	[24]
	Picrocrocin	—	Antiproliferative	[62]
	Picrocrocin	—	Anticancer	[63]
Monoterpenoids	Crocusatin D	—	Withdrawal syndrome, depression, spatial memory	[64]
	Crocusatin F			
	Crocusatin G			
	Crocusatin H			
	Crocusatin E			
	Crocusatin I			
Isophorones	Isomer of isophorone	Mice	Parkinson disease	[64]
	Isophorone		Hyperglycaemia–glucose uptake/metabolism	
General saffron extract	Crocin, crocetin, safranal, and picrocrocin	Mice	Anticancer effects, atherosclerosis, myocardial ischaemia, cardioprotection, anxiety, and insomnia	[64]

## Data Availability

All the data supporting the findings of this study are included in this article.
